# Survival of Black and White Patients With Stage IV Small Cell Lung Cancer

**DOI:** 10.3389/fonc.2021.773958

**Published:** 2021-12-10

**Authors:** Huashan Shi, Kexun Zhou, Jordan Cochuyt, David Hodge, Hong Qin, Rami Manochakian, Yujie Zhao, Sikander Ailawadhi, Alex A. Adjei, Yanyan Lou

**Affiliations:** ^1^ Department of Cancer Biology, Mayo Clinic, Jacksonville, FL, United States; ^2^ Department of Health Sciences Research/Biomedical Statistics and Informatics, Mayo Clinic, Jacksonville, FL, United States; ^3^ Division of Hematology and Medical Oncology, Mayo Clinic, Jacksonville, FL, United States; ^4^ Division of Medical Oncology, Mayo Clinic, Rochester, MN, United States

**Keywords:** stage IV Small cell lung cancer, racial, socioeconomic status, survival, academic program

## Abstract

**Background:**

Small cell lung cancer (SCLC) is associated with aggressive biology and limited treatment options, making this disease a historical challenge. The influence of race and socioeconomic status on the survival of stage IV SCLC remains mostly unknown. Our study is designed to investigate the clinical survival outcomes in Black and White patients with stage IV SCLC and study the demographic, socioeconomic, clinical features, and treatment patterns of the disease and their impact on survival in Blacks and Whites.

**Methods and Results:**

Stage IV SCLC cases from the National Cancer Database (NCDB) diagnosed between 2004 and 2014 were obtained. The follow-up endpoint is defined as death or the date of the last contact. Patients were divided into two groups by white and black. Features including demographic, socioeconomic, clinical, treatments and survival outcomes in Blacks and Whites were collected. Mortality hazard ratios of Blacks and Whites stage IV SCLC patients were analyzed. Survival of stage IV SCLC Black and White patients was also analyzed. Adjusted hazard ratios were analyzed by Cox proportional hazards regression models. Patients’ median follow-up time was 8.18 (2.37-15.84) months. Overall survival at 6, 12, 18 and 24 months were 52.4%, 25.7%, 13.2% and 7.9% in Blacks in compared to 51.0%, 23.6%, 11.5% and 6.9% in Whites. White patients had significantly higher socioeconomic status than Black patients. By contrast, Blacks were found associated with younger age at diagnosis, a significantly higher chance of receiving radiation therapy and treatments at an academic/research program. Compared to Whites, Blacks had a 9% decreased risk of death.

**Conclusion:**

Our study demonstrated that Blacks have significant socioeconomic disadvantages compared to Whites. However, despite these unfavorable factors, survival for Blacks was significantly improved compared to Whites after covariable adjustment. This may be due to Blacks with Stage IV SCLC having a higher chance of receiving radiation therapy and treatments at an academic/research program. Identifying and removing the barriers to obtaining treatments at academic/research programs or improving the management in non-academic centers could improve the overall survival of stage IV SCLC.

## Introduction

Blacks bear a disproportionate burden of cancers. Blacks have the lowest survival rate and the highest death rate in comparison to other racial or ethnic groups for most cancer types (1). Socioeconomic status (SES), such as income status, education level, and medical insurance, plays the most critical role in leading to these racial inequalities (1–5). Blacks have a higher rate with stage IV cancer, and the risk of cancer-related death is higher when compared with Whites because more Blacks are uninsured than Whites (6–11). Previous studies reported that the 5-year survival rate of lung cancer is lower in Blacks than those in Whites (1). In addition to socioeconomic status, curative-intent surgery also plays a vital role in survival. Blacks diagnosed with early-stage lung cancer are less likely to perform radical surgery than Whites even after considering the impact of socioeconomic factors (1, 12, 13). While the roles of race and SES disparity have been well-studied in various cancer care settings, their roles and interplay in stage IV SCLC remains primarily unknown. SCLC accounts for 10-15% of all lung cancers, and the prognosis for SCLC patients is poor (14–16). More than 60% of SCLC patients present with stage IV disease at diagnosis (17, 18). Differences in lung cancer incidence still exist among different ethnic groups, with Blacks having a significantly higher lung cancer rate than Whites and Blacks who are diagnosed with more stage IV cancers than Whites (1). In comparison, the incidence of SCLC decreases among all races, and there is no significant difference in stage distributions between Blacks and Whites from 2006 to 2010 (19).

NCDB is a prospectively maintained registry database covering 70% of newly diagnosed cancer cases in the nation (20). It includes 82% of lung cancer cases with an annual follow-up of at least 90% of the patients (21). In this current study, we analyzed the survival differences in stage IV SCLC patients between Blacks and Whites, considering various variables available in NCDB. We also investigated the characteristics of various clinical and treatment-related features among Blacks and Whites and the potential impact on clinical outcomes.

## Methods

SCLC cases diagnosed between 2004 and 2014 were obtained from the National Cancer Database (22). The histology codes were mainly based on the International Agency for Research on Cancer (IARC) classifications: small cell carcinoma [International Classification of Diseases for Oncology Third Edition (ICD-O-3) codes 8002, 8041–8045)]. We identified 214,096 cases with TNM staging data, excluding stage I, II, III, and the stage unknown cases. The final study cohort consisted of 119,611 stage IV SCLC patients, including 110,696 White patients and 8,915 Black patients.

Study subjects were included in the study from the date of diagnosis and were followed until the end of the study period, the date of the last contact, or death, whichever came first. The primary outcome measure in our study was overall survival (OS) (20). Baseline demographic features including sex, age, education, census median income quartiles, insurance, living area, geographic region, distance to treating facility (great circle distance, distance in miles between patient’s residence based on ZIP code centroid or city to street address of treating facility); clinical characteristics including the time of diagnosis, tumor size, Charlson-Deyo score, and treatments including radiation therapy, chemotherapy, immunotherapy, palliative care, facility procedure volume, academic/research program between Blacks and Whites were studied. Patients with missing information were excluded from the analysis. Predictors included age (<60, 60-69, 70-79, >=80), insurance at the time of diagnosis (private, government, no insurance, missing), percentage of without high school degree 2007-2012 (<7%, 7-12.9%, 13-20.9%, >=21%), census median income quartile 2007-2012 (<$38,000, $38,000-$47,999, $48,000-$62,999, >$63,000) and Charlson-Deyo comorbidity score (0, 1, 2, >=3). A modified Charlson-Deyo score was calculated from preexisting comorbidities, which up to six conditions (23).

### Statistical Analysis

The distribution of demographics, clinical, and treatment features was compared between Blacks *vs*. Whites using Pearson’s chi-square test and Wilcoxon rank-sum test, as appropriate. Overall survival was calculated as the time from the initial diagnosis of stage IV SCLC to date of death or the last known alive. Kaplan-Meier analysis was used to compare survival differences between Blacks and Whites. Multivariable Cox regression modeling was used to identify independent features associated with survival in patients with stage IV SCLC and hazard ratios (HR) for mortality were presented (22). Nonproportional variables were used as stratification variables, and the proportional hazards assumptions were performed using Schoenfield residuals in the final analysis. The primary endpoint of the study was the survival difference between Whites and Blacks. The possible association of other features with survival was the secondary analysis. A two-sided P-value of less than 0.05 with a confidence interval limit at 95% was considered as statistical significance.

## Results

A total of 119,611 stage IV SCLC patients, including 110,696 White patients and 8,915 Black patients, were included in this study. The median follow-up is 8.18 months (range 2.37-15.84 months). Patients’ demographic, clinical, and treatment features were compared between Blacks and Whites (**Tables 1**–**3**). Blacks were more likely to be male, younger, with lower education and lower annual income than Whites on the logistic regression model. They also had shorter travel distances to their treatment site. The median travel distance between the patient’s primary residence and treatment site was 13.8 *vs*. 24.9 miles in Blacks *versus* Whites (P<0.0001). Blacks were found to have larger tumors (mean tumor size 58 *vs*. 54.2 mm, p <0.0001) and were more likely to receive radiation therapy (41.6% *vs*. 38.9%, p<0.0001) and treatment in academic centers (41.4% *vs*. 24.7%, p<0.0001) than Whites. In addition, Blacks were less likely to receive palliative therapy than Whites (20.7% *vs*. 21.7%, p = 0.03). Among stage IV SCLC patients who received the treatments, 7.7% of Blacks and 6.3% of Whites received radiation therapy alone (p<0.0001), 36.2% of Blacks and 38.2% of Whites received chemotherapy alone (p<0.0001), and 33.3% of Blacks and 32% of Whites received both chemotherapy and radiation therapy (p<0.0001, **Supplementary Table 1**). Among the patients who received radiation therapy and chemotherapy, 60.5% of Black and 62.6% White patients received chemotherapy first, followed by radiation therapy (p = 0.0299). In addition, more Blacks were found brain metastasis than Whites (27.7% *vs.* 24.6%, p<0.0001). The incidence of stage IV SCLC increased from 2004 to 2014 in both Blacks and Whites, although the distribution was slightly different. No significant differences were found between Blacks and Whites in insurance status, living area, geographic region, diagnostic confirmation method, Charlson-Deyo score, chemotherapy, immunotherapy, and treatment facility volume.

**Table 1:** Demographic characteristics of patients with stage IV SCLC.

	White (N = 110696)	Black (N = 8915)	*P* value
**Sex**			0.0147
Male	56673 (51.2%)	4606 (51.7%)	
Female	54023 (48.8%)	4309 (48.3%)	
**Age**			0.0145
<60	26653 (24.1%)	2586 (29.0%)	
60-69	38088 (34.4%)	3061 (34.3%)	
70-79	32960 (29.8%)	2386 (26.8%)	
>=80	12995 (11.7%)	882 (9.9%)	
**Percent No High School Degree 2007-2012^a^**			0.0122
Missing	2292	140	
>=21%	18633 (17.2%)	3433 (39.1%)	
13-20.9%	32178 (29.7%)	3289 (37.5%)	
7-12.9%	37236 (34.3%)	1561 (17.8%)	
<7%	20357 (18.8%)	492 (5.6%)	
**Census Median Income Quartiles 2007-2012^b^**			0.0034
Missing	2344	145	
<$38,000	20776 (19.2%)	4458 (50.8%)	
$38,000-$47,999	29978 (27.7%)	1964 (22.4%)	
$48,000-$62,999	30520 (28.2%)	1465 (16.7%)	
$63,000+	27078 (25.0%)	883 (10.1%)	
**Patient’s Insurance**			0.2267
Missing	4618	554	
No insurance	2220 (2.1%)	243 (2.9%)	
Government Insurance	73253 (69.1%)	6201 (74.2%)	
Private insurance	30605 (28.9%)	1917 (22.9%)	
**Type of area**			0.2763
Missing	4289	215	
Urban	20207 (19.0%)	781 (9.0%)	
Metro	83254 (78.2%)	7814 (89.8%)	
Rural	2946 (2.8%)	105 (1.2%)	
**Great Circle Distance**			<0.0001
N	108440	8763	
Mean (SD)	24.9 (91.6)	13.8 (53.9)	
Median	9.2	5.0	
**Geographic region**			0.1088
Missing	339	39	
East Coast	45258 (41.0%)	4225 (47.6%)	
Central	52345 (47.4%)	4223 (47.6%)	
Mountain	4087 (3.7%)	64 (0.7%)	
Pacific	8667 (7.9%)	364 (4.1%)	

^a,b^Variables refer to the residential region, rather than individual.

**Table 2:** Disease characteristics of patients with stage IV SCLC.

	White (N = 110696)	Black (N = 8915)	*P* value
**Year of Diagnosis**			<0.0001
2004	8393 (7.6%)	645 (7.2%)	
2005	8543 (7.7%)	649 (7.3%)	
2006	8838 (8.0%)	643 (7.2%)	
2007	8947 (8.1%)	708 (7.9%)	
2008	9933 (9.0%)	758 (8.5%)	
2009	10032 (9.1%)	836 (9.4%)	
2010	10816 (9.8%)	914 (10.3%)	
2011	11005 (9.9%)	912 (10.2%)	
2012	11200 (10.1%)	921 (10.3%)	
2013	11350 (10.3%)	971 (10.9%)	
2014	11639 (10.5%)	958 (10.7%)	
**Diagnostic confirmation**			0.3776
Missing	103	7	
Positive histology	92351 (83.5%)	7239 (81.3%)	
Positive cytology	18242 (16.5%)	1669 (18.7%)	
**Tumor size (mm)**			<0.0001
N	68419	5580	
Mean (SD)	54.2 (51.6)	58.0 (50.8)	
Median	47.0	50.0	
**Charlson-Deyo Score**			0.1180
0	61000 (55.1%)	4883 (54.8%)	
1	33422 (30.2%)	2522 (28.3%)	
2	11645 (10.5%)	1035 (11.6%)	
>=3	4629 (4.2%)	475 (5.3%)	

**Table 3:** Clinical treatment of patients with stage IV SCLC.

	White (N = 110696)	Black (N = 8915)	*P* value
**Radiation Therapy**			<0.0001
Missing	552	38	
No	67347 (61.1%)	5184 (58.4%)	
Yes	42797 (38.9%)	3693 (41.6%)	
**Chemotherapy**			0.1874
Missing	1512	146	
No	31134 (28.5%)	2559 (29.2%)	
Yes	78050 (71.5%)	6210 (70.8%)	
**Immunotherapy**			0.3848
Missing	352	48	
No	110019 (99.7%)	8846 (99.8%)	
Yes	325 (0.3%)	21 (0.2%)	
**Palliative Care**			0.0331
Missing	502	33	
No	86268 (78.3%)	7040 (79.3%)	
Yes	23926 (21.7%)	1842 (20.7%)	
**Facility Procedure Volume**			0.1189
Low	8706 (7.9%)	500 (5.6%)	
Medium	49572 (44.8%)	3792 (42.5%)	
High	52418 (47.4%)	4623 (51.9%)	
**Academic/Research Program**			<0.0001
Missing	339	39	
No	83049 (75.3%)	5201 (58.6%)	
Yes	27308 (24.7%)	3675 (41.4%)	

**Figure 1:**
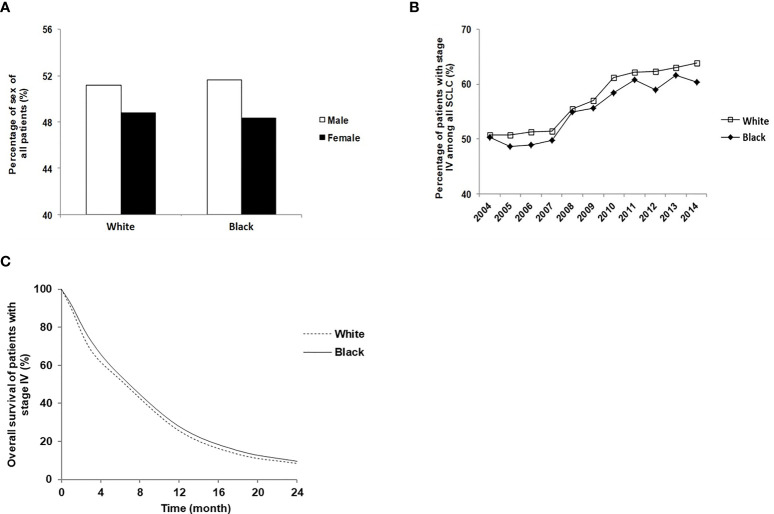
Disease distribution characteristics and survival time of stage IV SCLC patients of Black and White. **(A)** Sex distribution of White and Black stage IV SCLC patients; **(B)** The percentage of White and Black patients who were diagnosed with stage IV SCLC between 2004 and 2014; **(C)** The overall survival of White and Black stage IV SCLC patients.

Although the overall distribution was consistent regarding the patients’ and tumor’s characteristics, some differences were noticed. Among those with stage IV SCLC, the proportion of female patients was lower than male patients in both Whites (48.8% versus 51.2%) and Blacks (48.3% *versus* 51.7%) (**Figure 1A**). The total number of White stage IV SCLC patients increased by 27.9% in 2014 compared to 2004 (8,393 patients in 2004 and 11,639 patients in 2014). The number of Black stage IV SCLC patients increased by 32.7% in 2014 compared to 2004 (645 in 2004 and 958 in 2014). The percentage of stage IV SCLC among all patients with SCLC had also increased significantly from 2004 to 2014 in both Whites and Blacks, and the increases in Whites were more than those in Blacks (**Figure 1B**). The median overall survival time of stage IV SCLC in Blacks was 6.57 months, higher than 6.21 months in Whites (p<0.001). The 6 months, 12 months, 18 months and 24 months’ survivals were 51.0%, 23.6%, 11.5%, 6.9% for Whites and 52.4%, 25.7%, 13.2%, 7.9% for Blacks (p<0.01) (**Figure 1C**).

**Figure 2:**
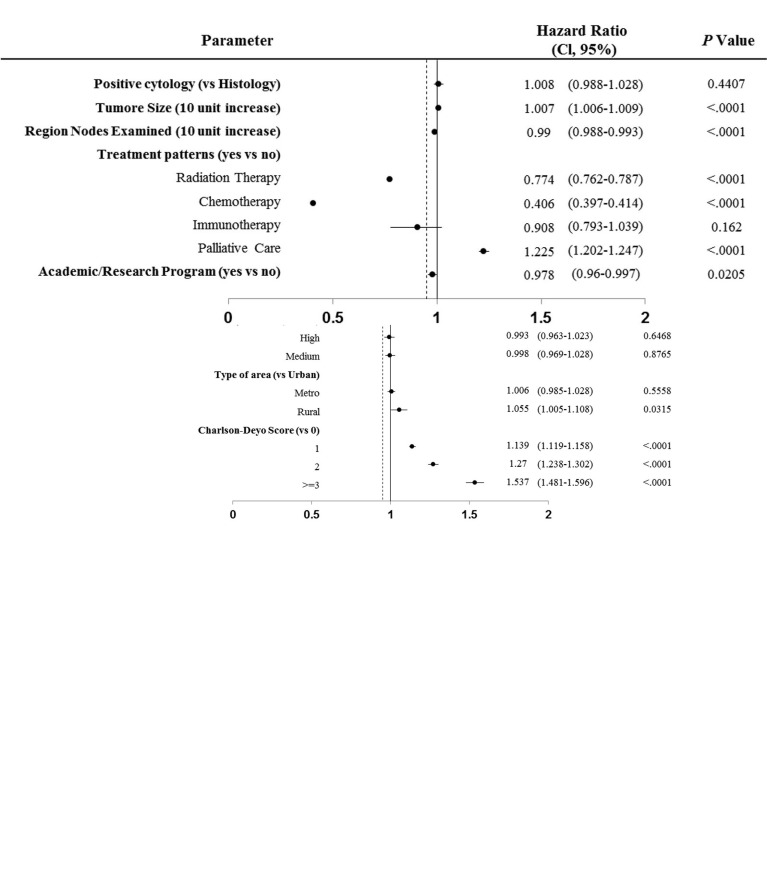
Cox proportional hazards multivariable regression analysis for predictors of overall survival of patients with stage IV SCLC.

We next used the Cox proportional hazards multivariable model to analyze the potential predictors of overall survival of patients with stage IV SCLC. Black was independently associated with a decreased hazard of death in Cox proportional hazards modeling after controlling for demographic and clinical factors (HR=0.911; 95%CI: 0.884-0.938; P<0.0001). Other favorable factors associated with improved OS included female (HR=0.852; 95% CI: 0.839-0.864, P<0.0001), greater distance to treatment center (HR=0.988; 95%CI: 0.983-0.993, P<0.0001), higher income particularly income>63,000 (HR=0.929; 95% CI: 0.901-0.958, P<0.0001), private insurance (HR=0.888; 95% CI: 0.835-0.945, P<0.0001), radiation therapy (HR=0.774; 95% CI: 0.762-0.787, P<0.0001), chemotherapy (HR=0.406; 95%CI:0.397-0.414, P<0.0001), and receiving treatment in academic/research center (HR=0.978; 95% CI: 0.96-0.997, P=0.02) (**Figure 2**). Treatment with Chemotherapy appeared to be the most favorable predictor of survival in patients with stage IV SCLC. By contrast, factors associated with decreased OS included increase in age (HR=1.136; 95% CI: 1.126-1.147, P<0.0001), high education (HR=1.05; 95% CI: 1.017-1.085, P=0.0029), living in Rural (HR=1.055; 95% CI: 1.005-1.108, P = 0.0315), high Charlson-Deyo score (HR=1.537; 95% CI: 1.481-1.596, P < 0.0001), increase in tumor size (HR=1.007; 95% CI: 1.006-1.009, P<0.0001) and palliative care (HR=1.225; 95% CI: 1.202-1.247, P<0.0001).

## Discussion

Cancer screening, advances in surgery and radiation techniques, and developments of novel therapeutic agents have undoubtedly led to improved clinical outcomes in many cancer patients. However, new developments and advances are uneven among different cancer types, and disparities exist in clinical outcomes across multiple cancer types and some attributes to modifiable factors. Stage IV SCLC represented one of the most notorious cancers with only 2-4 months of survival in untreated patients. Chemotherapy using platinum‐based doublet remains a cornerstone of first-line treatment until the very recent breakthrough of adding the benefit of immunotherapy in combination with chemotherapy (24, 25). Although modest improvement in survival has been observed in patients with SCLC over time, the differences in clinical outcome, socioeconomic status, clinical features, and treatment patterns between races, particularly Blacks *versus* Whites, remain mostly unknown. Our current study represents the largest real-world analysis of stage IV SCLC patients to investigate the clinical outcomes, the impacts of SES, clinical and treatment factors among Blacks and Whites.

Multiple studies have examined the impact of race and ethnicity on overall lung cancer prognosis (26–28). These studies have demonstrated that non-Black patients have had favorable survival than Blacks over the last several decades. In fact, Blacks have been reported to have the lowest 1-year, and 3-year survival rates in patients with non-small cell lung cancer among all races (29), and the 5-year survival rate for lung cancer is lower in Blacks than in Whites (16% *vs*. 19%, respectively) (1). In a multivariate analysis of SCLC patients treated through Southwest Oncology Group trials, Whites were found associated with a more favorable clinical outcome than Blacks (26). A few factors such as differences in treatments, time of diagnosis, and possible biological variability were suspected of contributing to the difference observed in Whites *versus* non-Whites. By contrast, using the CALGB lung cancer database, Stock et al. found that the overall survival of Black patients was not significantly different from that of non–Black patients with or without adjustment for histology, treatment, and metastatic site, despite Black patients were more likely to present with worse performance status and lower socioeconomic condition (30). Both studies offered high-quality information built upon the constraints of clinical trial entry criteria, close follow-up, and relatively uniform treatment regimens through the studies. However, the overall numbers of SCLC patients remain relatively small, and the patients who enter the clinical studies may not best represent patients in the real world. Our study specifically focused on stage IV SCLC where the treatment regimens have been relatively uniform to control the potential confounding effects of stages on outcomes. Our study included a total of 119,611 stage IV SCLC patients. We demonstrated that Blacks with stage IV SCLC had improved overall survival compared to Whites (HR 0.91, CI: 0.884-0.938; P<0.0001). This finding is interesting as our analysis revealed that Blacks were more likely to be associated with unfavorable factors such as lower income levels and larger tumor size. Despite the lower socioeconomic status and larger tumor, Blacks’ one-year and two-year survival were qualitatively similar and statistically superior to Whites (25.7% *versus* 23.6% and 7.9% *versus* 6.9% respectively, p<0.01). This might be because more Black patients received radiation therapy (41.6% *vs*. 38.9%, p<0.0001), had a younger age at diagnosis, and received treatments in academic/research programs (41.4% *vs*. 24.7%, p<0.0001) than Whites.

Consolidative thoracic radiotherapy and prophylactic cranial irradiation (PCI) have been recommended in patients with stage IV SCLC based on improved overall survival (15, 31). Particularly, PCI has been recommended as the standard of care during our study period, although a recent study performed in Japan demonstrated inconsistent conclusions. Nevertheless, our data found that radiation therapy led to a 23% reduction in mortality (HR 0.77, CI: 0.762-0.787; P<0.0001) in patients with stage IV, and remained the second most favorable factor. It was not surprising to see that chemotherapy represented the most favorable factor in patients with stage IV SCLC (HR 0.41, CI: 0.397-0.414; P<0.0001). Our data suggested that the improved overall survival observed in Blacks than Whites might be related to more Blacks receiving radiation therapy than Whites. Unfortunately, we are unable to classify further the type of radiation (to the primary site *vs* prophylactic) and location of radiation therapy (thoracic, brain, or other organs) due to limitations of the NCDB dataset. Further studies in this regard are warranted and may be done through claims-based databases. Age was found to be an unfavorable prognosis factor in our study (HR 1.14, CI: 1.126-1.147; P<0.0001), and Blacks were more likely to be diagnosed at an age younger than 60 years (29% *vs*. 24.1%, p=0.0145). This finding is consistent with our previous findings in non-small cell lung cancer (20). It is also found Black patients have superior survival compared to White patients with multiple myeloma, particularly due to diagnosis in the younger population (22, 32).

One of the most striking differences we noticed between Blacks and Whites was that Blacks were more likely to receive their treatments in academic/research programs than Whites. In our study, 41.4% of Black patients with stage IV SCLC received treatment at an academic/research program in contrast to 24.7% in White patients. Our study also demonstrated that treatment in academic/research centers was a favorable factor, therefore likely contributing to the improved survival in Blacks that we observed in our study. This data suggested receiving care at an academic/research center might mitigate the significant SES disadvantages in society and medical care as an actionable approach. This was similar to our previous study in NSCLC where we found the initial therapy at academic centers significantly improved clinical outcomes (20). Similarly, treatment at academic centers was also demonstrated independently associated with improved survival in patients with locally advanced head and neck cancer (22, 33). The underlying factors driving the improved outcomes are likely multifactorial. Academic/research centers more likely provide access to clinical trials, multi-disciplinary expertise, and ancillary services. Unfortunately, we cannot determine each factor’s exact impact on the survival of stage IV SCLC patients in our study due to the lack of such information in NCDB (34).

Consistent with previous studies performed in non-small cell lung cancers, our study demonstrated that Blacks were associated with lower socioeconomic status, including lower education and low annual incomes than Whites (20, 35–39). Interestingly, high education was found to not correlate with improved clinical outcomes. Instead, it was associated with worse clinical outcomes, although the difference was small (HR 1.05, CI: 1.017-1.085; P=0.0029). This is different from findings from other cancers, including non-small cell lung cancer, where a high level of education has been a beneficial factor for survival (34, 40, 41). This may be due to the ratio of no high school degree patients referred to the residential region rather than individual. Another reason might be that most Blacks are less educated and have better outcomes. Patients with high annual income, particularly those with > $ 63,000, were associated with favorable outcomes, likely due to more access to treatments. Similarly, females were also found to have better outcomes in our study, consistent with findings from other cancer types (39).

Not surprisingly, high comorbidity status was found closely correlated with poor prognosis. Palliative care was found to be associated with poor prognosis in our study. This may be because patients who received palliative care were more symptomatic and had lower tolerability to therapy. It has been noted previously that palliative care utilization is extremely low even amongst academic cancer centers, suggesting that its use is more associated with an alternative than an adjunct to active anticancer therapeutics (42). During this period of 2004 to 2014, the incidence of SCLC declined (43). However, the percentages of stage IV SCLC were increased in both White and Blacks, indicating potentially later diagnosis of this disease.

### Limitations

While our study was the first large-scale data analysis focusing on various factors associated with stage IV SCLC outcomes in Blacks and Whites, several limitations were noticed. This study was performed retrospectively through NCDB database, and therefore selection bias was presented. As shown in our study, Blacks with stage IV SCLC had significant disadvantages in socioeconomic status compared to Whites. Thus, Blacks with stage IV SCLC were more likely to present without complete staging information and might be under-represented in our study. The imbalance in the numbers of Blacks and Whites was also evident. However, the number of Blacks included in this study is still higher than other available databases, such as The Surveillance, Epidemiology, and End Results (SEER) Program. Furthermore, although the NCDB contains relatively comprehensive information on cancer patients in the United States, some detailed information was not available. For example, the details of individual treatment, chemotherapy regimens and the number of cycles, accurate radiation dose and field, and comorbidities were not available, which might impact assessment accuracy. Besides the treatment status, it is also a lack of the smoking data of patients, which is important for the survival of SCLC patients. In addition, due to the dismal prognosis associated with stage IV SCLC, the magnitude of overall survival difference between Blacks and Whites is relatively small.

## Conclusions

Our study demonstrated that Blacks were associated with significant socioeconomic disadvantages in comparison with Whites. However, despite these unfavorable factors, Blacks had survival outcomes qualitatively similar and statistically superior than those of Whites after co-variable adjustment. Blacks were found associated with younger age at diagnosis, a significantly higher chance of receiving radiation therapy and treatments at the academic/research program. This suggests the importance of radiotherapy and receiving care at an academic center could mitigate the known SES disadvantages in treating small cell lung cancer patients.

## Data Availability Statement

The datasets presented in this study can be found in online repositories. The names of the repository/repositories and accession number(s) can be found below: National cancer database.

## Ethics Statement

The studies involving human participants were reviewed and approved by Mayo Clinic IRB. Written informed consent for participation was not required for this study in accordance with the national legislation and the institutional requirements.

## Author Contributions

HS and YL drafted the article. HS, KZ, and YL designed the study. JC and DH helped the statistical analysis. HS, KZ, HQ, AA, RM, YZ, AS, and YL give critical revision of the article for important intellectual content. All authors contributed to the article and approved the submitted version.

## Funding

This work was supported by the National Institutes of Health [grant number is K12CA090628, YL].

## Conflict of Interest

YL: Advisory board: AstraZeneca, Novocure; Consultant: AstraZeneca; Honorarium: clarion health care; Research Funding Support: Merck, MacroGenics, Tolero Pharmaceuticals, AstraZeneca, Vaccinex, Blueprint Medicines, Harpoon Therapeutics, Sun Pharma Advanced Research, Bristol-Myers Squibb, Kyowa Pharmaceuticals, Tesaro, Bayer HealthCare. RM: Advisory board: AstraZeneca, Guardant Health, Novocure, Takeda; Consulting: AstraZeneca. AS is a consultant with Celgene, Takeda, Janssen, Amgen, AstraZeneca, Glaxo-Smithkline, and has received institutional research support from Pharmacyclics, Bristol Myers Squibb, Janssen, Amgen, Cellectar, Medimmune, and Ascentage.

The remaining authors declare that the research was conducted in the absence of any commercial or financial relationships that could be construed as a potential conflict of interest.

## Publisher’s Note

All claims expressed in this article are solely those of the authors and do not necessarily represent those of their affiliated organizations, or those of the publisher, the editors and the reviewers. Any product that may be evaluated in this article, or claim that may be made by its manufacturer, is not guaranteed or endorsed by the publisher.

## Supplementary Material

Supplementary Table 1 Chemotherapy/radiation treatment and brain metastasis in patients with stage IV SCLC

## Abbreviations

SCLC, small cell lung cancer; NCDB, National Cancer Database; SES, Socioeconomic status; IARC, International Agency for Research on Cancer; OS, overall survival; PCI, prophylactic cranial irradiation; SEER, The Surveillance, Epidemiology, and End Results Program.
