# Supplementary material for: Survival of Black and White Patients With Stage IV Small Cell Lung Cancer
Source: Front Oncol. 2021 Dec 10;11:773958. doi: 10.3389/fonc.2021.773958 (PMC8702563; doi:10.3389/fonc.2021.773958)
Supplement: Supplementary Table 1 — Chemotherapy/radiation treatment and brain metastasis in patients with stage IV SCLC [file Table_1.pdf]

Supplementary Table 1

	NH Black (N=8915)	NH White (N=110696)	Total (N=119611)	p value
**Chemotherapy + Radiation**				0.0120
No	5944 (66.7%)	75235 (68.0%)	81179 (67.9%)	
Yes	2971 (33.3%)	35461 (32.0%)	38432 (32.1%)	
**Chemotherapy only**				0.0001
No	5692 (63.8%)	68399 (61.8%)	74091 (61.9%)	
Yes	3223 (36.2%)	42297 (38.2%)	45520 (38.1%)	
**Radiation only**				<0.0001
No	8230 (92.3%)	103730 (93.7%)	111960 (93.6%)	
Yes	685 (7.7%)	6966 (6.3%)	7651 (6.4%)	
**Sequence of Chemotherapy and radiation**				0.0299
1st Chemotherapy, 2nd radiation	1590 (60.5%)	19394 (62.6%)	20984 (62.5%)	
1st Radiation, 2nd Chemotherapy	1038 (39.5%)	11568 (37.4%)	12606 (37.5%)	
**Metastatic Brain Involvement**				<0.0001
No	2666 (72.3%)	33737 (75.4%)	36403 (75.2%)	
Yes	1019 (27.7%)	10992 (24.6%)	12011 (24.8%)	
